# Hip fractures among the elderly in Kyoto, Japan: a 10-year study

**DOI:** 10.1007/s11657-021-00888-8

**Published:** 2021-02-12

**Authors:** Maki Asada, Motoyuki Horii, Kazuya Ikoma, Tsuyoshi Goto, Naoki Okubo, Nagato Kuriyama, Kenji Takahashi

**Affiliations:** 1 Rakuwakai Otowa Rehabilitation Hospital, Kyoto, Japan; 2 Department of Orthopaedics, Graduate School of Medical Science, Kyoto Prefectural University of Medicine, Kyoto, Japan; 3 Department of Rehabilitation Medicine, Graduate School of Medical Science, Kyoto Prefectural University of Medicine, Kyoto, Japan; 4 Department of Epidemiology for Community Health and Medicine, Graduate School of Medical Science, Kyoto Prefectural University of Medicine, Kyoto, Japan

**Keywords:** Hip fracture, Femoral neck fracture, Trochanteric fracture, Incidence

## Abstract

***Summary*:**

In Kyoto Prefecture, Japan, the number of hip fractures increased during 2013–2017 compared to 2008–2012. However, the estimated overall incidence rate increased only in femoral neck fractures in men aged ≥75 and women aged ≥85.

**Purpose:**

The incidence rate of hip fractures in Japan has plateaued or decreased. We investigated the annual hip fracture occurrences in Kyoto Prefecture, Japan, from 2008 to 2017.

**Methods:**

Patients aged 65 years and above who sustained hip fractures between 2008 and 2017 and were treated at one of the participating 11 hospitals were included. The total number of beds in these institutions was 3701, accounting for 21.5% of the 17,242 acute-care beds in Kyoto Prefecture. The change in incidence rate was estimated utilizing the population according to the national census conducted in 2010 and 2015.

**Results:**

The total number of hip fractures was 10,060, with 47.5% femoral neck fractures and 52.5% trochanteric fractures. A decrease in number was seen only in trochanteric fractures in the group of 75- to 84-year-old women. The population-adjusted numbers of femoral neck fractures showed a significant increase in all age groups in men, whereas in women, there was an increase in femoral neck fractures in the ≥85 group and trochanteric fractures in the age group 65–74, and a decrease in trochanteric fractures in the age group 75–84. The estimated change in incidence rate showed an increase in femoral neck fractures in men aged ≥75 and women aged ≥85.

**Conclusion:**

In Kyoto Prefecture, the number of hip fractures increased in the second half of the study period (2013–2017) compared to the first half (2008–2012). However, the incidence rate had not increased, except in femoral neck fractures in men aged ≥75 and women aged ≥85.

## Introduction

The number of hip fractures continues to grow in Japan with the aging population. However, the incidence rate has been reported to have slowed down in growth and decreased in both men and women aged 70–79 years [1]. A decrease in the incidence rate of hip fractures has also been noted in many Western countries.

Hip fractures can be divided into femoral neck fractures which are intraarticular and trochanteric fractures which are extraarticular. Literature shows that discrepancies in their incidence rates exist with differences in geography, age, height, bone density, and vertebral fracture complication rates [2]. Hagino et al. [3] reported a recent increase in the ratio of patients with femoral neck fractures to those with trochanteric fractures (the N/T ratio).

In order to improve prevention strategies for hip fractures in the prefecture of Kyoto Japan, where the incidence rate of hip fractures is more than 1.2 times that of the national rate [1], we started a 10-year study on hip fractures using registration forms in accordance with the nationwide survey of the Japanese Orthopaedic Association (JOA) [4]. We hypothesized that hip fractures in Kyoto would also show a decrease in the rate of growth.

## Materials and methods

This was a retrospective, multicenter, observational study. Eleven JOA-authorized hospitals in Kyoto Prefecture, Japan, were involved. The total number of beds in these institutions were 3701, accounting for 21.5% of the 17,242 acute-care beds in Kyoto Prefecture.

Patients aged 65 years or older who sustained hip fractures between January 1, 2008, and December 31, 2017, and were treated at one of the participating hospitals were included. Patients with isolated greater trochanteric, subtrochanteric, or pathologic fracture were excluded.

The following data were obtained using registration forms from a JOA nationwide survey [4, 5]: sex, age, and fracture type (femoral neck vs. trochanteric). The diagnoses were made by board-certified orthopedic surgeons. Ages were categorized as 65–74, 75–84, or ≥85 years.

The study period was divided into the first 5 years (2008–2012), hereafter referred to as the first half, and the last 5 years (2013–2017) referred to as the second half. The numbers of femoral neck fractures and trochanteric fractures were compared between the two periods by sex as well as age group.

**Fig. 1:**
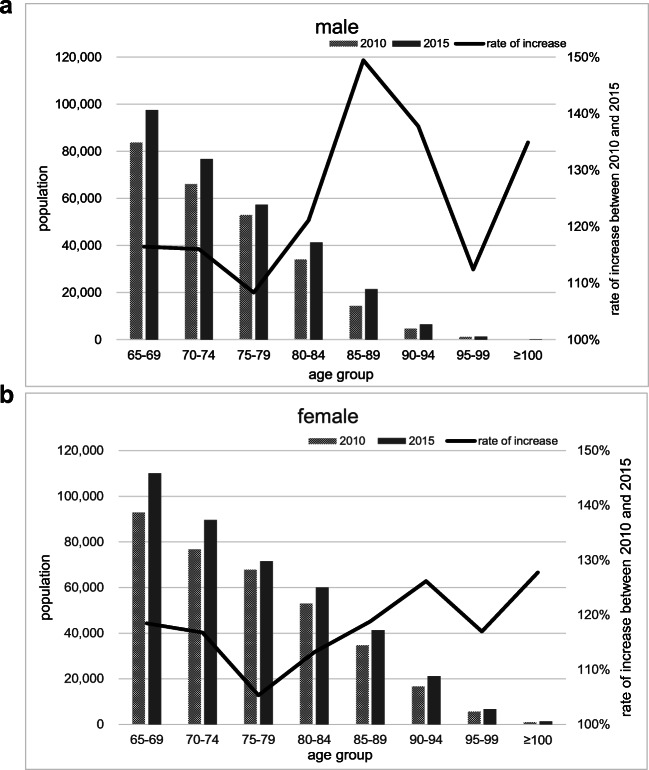
**a**, **b** The change in the population over 65 in Kyoto Prefecture between 2010 and 2015. The curve shows the rate of increase or decrease calculated by the population in 2015 divided by that in 2010.

In addition, to estimate the rate of change in incidence, the population-adjusted number of hip fractures (adjusted number) in the second half of the period was calculated, applying the demographics in the 2010 and 2015 census (Fig. 1) as the population of the first half and second half. To be specific, the number of hip fractures in the second half was divided by the ratio of the change in population between the first and second half. Then the number of fractures in the first half was divided by this adjusted number of fractures in the second half in each age group, in order to estimate the rate of change in incidence from the first to the second half of the study period. These population adjustments were made using the actual incidence and demographic changes for every 5 years of age. Using the hip fracture data distributed annually by the Japanese Orthopedic Association, we calculated the nationwide N/T ratio for reference in the first and second half of this study.

Ethical approval was obtained from the ethics committee of the Kyoto Prefectural University of Medicine.

The statistical comparison was made using the Poisson distribution, and a *p* value of 0.05 or less was considered a significant difference. The chi-squared exam was used to compare the N/T ratio. Statistical analyses were conducted with StatFlex ver 6.0 (Artech Co., Ltd., Osaka, Japan).

## Results

The total number of hip fractures was 10,060, with 4776 (47.5%) femoral neck fractures and 5284 (52.5%) trochanteric fractures. The percentages of women were 79.9% in femoral neck fractures and 80.0% in trochanteric fractures. Table 1 shows the number of hip fractures in the first and second half. Only trochanteric fractures in the 75- to 84-year-old group in women had decreased in number.

**Table 1:** The number of hip fractures and N/T ratio in the first and second half of the study period. Incidences in each sex and age group are shown. Significant increases/decreases in the second 5 years are shown by bold type. N/T signifies the rate of femoral neck fractures divided by trochanteric fractures

	Age group	Hip fracture			Femoral neck fracture			Trochanteric fracture			N/T**		
		First*	Second	*p*	First	Second	*p*	First	Second	*p*	First	Second	*p*
Male	65–74	**150**	**205**	0.000	**71**	**120**	0.000	79	85	0.500	**0.899**	**1.412**	0.037
	75–84	**390**	**484**	0.000	**172**	**252**	0.000	218	232	0.343	**0.789**	**1.086**	0.019
	≥85	**304**	**480**	0.000	**116**	**227**	0.000	**188**	**253**	0.000	**0.617**	**0.897**	0.012
Female	65–74	**394**	**511**	0.000	**273**	**337**	0.000	**121**	**174**	0.000	2.256	1.937	0.288
	75–84	1387	1394	0.851	746	784	0.164	641	610	0.221	1.164	1.285	0.193
	≥85	**1920**	**2414**	0.000	**691**	**960**	0.000	**1229**	**1454**	0.000	**0.562**	**0.660**	0.011
All	65–74	**544**	**716**	0.000	**344**	**457**	0.000	**200**	**259**	0.000	1.720	1.764	0.829
	75–84	**1777**	**1878**	0.017	**918**	**1036**	0.000	859	842	0.562	**1.069**	**1.230**	0.034
	≥85	**2224**	**2894**	0.000	**807**	**1187**	0.000	**1417**	**1707**	0.000	**0.570**	**0.695**	0.001

^*^“First” means the first half of the period (2008–2012) and “second” means the second half of the period (2013–2017)

^**^N/T, the ratio of patients with neck fractures to those with trochanteric fractures

Bold numbers indicate significant differences between the first and second half of the period (*p* < 0.05)

**Fig. 2:**
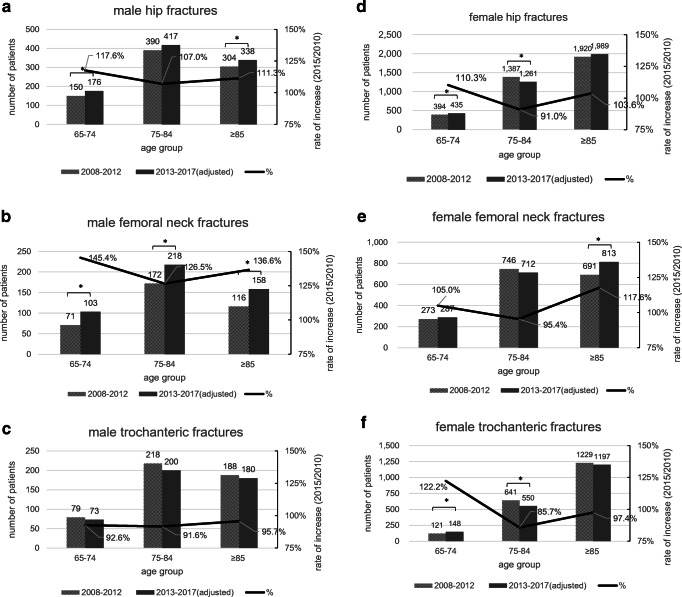
**a**–**f** Comparison of the number of hip fractures in the first and second half of the study period with adjustment for the population. The curve shows the rate of increase or decrease calculated by the population in 2015 divided by that in 2010. Asterisks show significant differences

An increase in population from 2010 to 2015 in all ages of 65 and above in both sexes was seen by plotting the population according to the national census (Fig. 1). Figure 2 shows the number of fractures in the first half and the population-adjusted number in the second half. The adjusted numbers of femoral neck fractures show a significant increase in all age groups in men. In women, the adjusted number of femoral neck fractures in the over-85 group and adjusted number of trochanteric fractures in the age group 65–74 increased, whereas the adjusted number of trochanteric fractures in the age group 75–84 decreased. The N/T ratio increased in the second half in all age groups in men, and in the over-85 group in women (Table 1). The nationwide N/T ratio in the first and second half of this study (Table 2) showed an increase in all age groups in both sexes.

**Table 2:** Comparison of the N/T ratio* in the first and second half of the period in nationwide Japan

Period**		First	Second
Male	65–74	1.181 (7709/6528)	1.392 (9955/7152)
	75–84	0.915 (14,853/16,236)	1.067 (19,189/17,992)
	≤85	0.746 (10,166/13,624)	0.823 (16,330/19,836)
Female	65–74	2.110 (21,160/10,029)	2.515 (25,389/10,094)
	75–84	1.060 (54,685/51,596)	1.178 (62,173/52,776)
	≤ 85	0.596 (53,138/89,198)	0.609 (71,611/11,7496)

^*^N/T ratio, the ratio of patients with femoral neck fractures to those with trochanteric fractures

^**^Period: “First” means the first half of the period (2008–2012) and “second” means the second half of the period (2013–2017)

The analysis was based on the hip fracture data distributed annually by the Japanese Orthopedic Association

The figures in parentheses indicate the actual number of occurrences

For all items, there are significant differences between the first and second half of the period (chi-square test *p* < 0.0005)

## Discussion

The incidence rates of hip fractures have been reported to be decreasing globally [6–9] as well as in Japan [1, 10, 11]. This trend has been attributed to promotion of health programs to prevent falls [7, 8, 12], changes in lifestyle [8], and the wider use of anti-osteoporotic drugs [1, 6, 8–11, 13].

Meanwhile, Hagino et al. [3] have reported the relative increase of femoral neck fractures in comparison to trochanteric fractures in Japan. The N/T ratio in the first and second half of this study showed a nationwide increasein all age groups in both sexes. Tamaki et al. reported in a study using the National Health Insurance Claim Database that the age-standardized hip fracture incidence rates indicated no significant change in females and a significant increase in males in Japan from 2012 to 2015 [14]. Gender differences in the trends in fracture incidence have also been noted, with a decrease in fracture incidence in females relative to males over the last 20 years [15].

Our study showed the number of hip fractures in Kyoto prefecture significantly increased in the second half of the study period in all groups except in women aged 75–84. In men, femoral neck fractures increased in all age groups, but trochanteric fractures increased only in the over-85 group. Although it is impossible to calculate the exact incidence rate in this study, by applying the change in the population, the change in incidence rate can be estimated. Taking demographics into account, the population-adjusted estimated number of hip fractures significantly decreased in women aged 75–84, largely due to the reduction in trochanteric fractures. On the other hand, the population-adjusted number of hip fractures in men did not decrease. By fracture type, trochanteric fractures did not increase, while femoral neck fractures increased significantly in each age group.

Our study indicated a difference in the trend of incidence by gender and fracture type. It also suggested that the current treatment and or prevention strategies being taken may be insufficient with regard to femoral neck fractures. Femoral neck fractures have been reported to occur in taller, younger individuals with higher bone densities and in more urban settings, compared to trochanteric fractures [2]. Because age had increased within the 10-year period, the change in the pattern of hip fractures in Kyoto could be a reflection of changes in any of the remaining factors. The study period coincides with the increased use of bisphosphonates in Japan, and although bisphosphonates have been shown to decrease hip fractures[16, 17], they are also known to increase bone density, and their generalized use may have had a greater preventive effect on trochanteric fractures compared to femoral neck fractures. Further investigation into the factors affecting hip fracture patterns in Kyoto should lead to more effective prevention strategies, taking into account bone quality for example.

One of the limitations of this study is that the actual incidence rate data could not be obtained. We tried to minimize this weakness by adjusting for a population for each 5-year age group in order to obtain a more accurate estimation of the rate of change in incidence. Another limitation is that confounding factors such as complications, history of corticosteroid use, and body figure are unknown. Needless to say, a coverage closer to 100% of the acute beds of the prefecture would have been preferable, rather than the 21.5% that we were able to obtain. On the other hand, all institutions involved were JOA-authorized hospitals, and data were collected by orthopedic surgeons, so a more accurate diagnosis of fracture type could be expected.

In conclusion, there was an increase in the number of hip fractures in Kyoto prefecture, Japan, in the second half of the study period (2013–2017) compared to the first half (2008–2012). However, the estimated overall increase in incidence rate was subdued, with the exception of femoral neck fractures in men aged 75 and above and women aged 85 and above which increased.
